# MyGeneset.info: an interactive and programmatic platform for community-curated and user-created collections of genes

**DOI:** 10.1093/nar/gkad289

**Published:** 2023-04-18

**Authors:** Ricardo Avila, Vincent Rubinetti, Xinghua Zhou, Dongbo Hu, Zhongchao Qian, Marco Alvarado Cano, Everaldo Rodolpho, Ginger Tsueng, Casey Greene, Chunlei Wu

**Affiliations:** Department of Integrative Structural and Computational Biology, The Scripps Research Institute, La Jolla, CA, USA; Department of Biochemistry and Molecular Genetics, Center for Health AI, University of Colorado School of Medicine, Aurora, CO, USA; Department of Integrative Structural and Computational Biology, The Scripps Research Institute, La Jolla, CA, USA; Department of Biochemistry and Molecular Genetics, Center for Health AI, University of Colorado School of Medicine, Aurora, CO, USA; Department of Integrative Structural and Computational Biology, The Scripps Research Institute, La Jolla, CA, USA; Department of Integrative Structural and Computational Biology, The Scripps Research Institute, La Jolla, CA, USA; Department of Integrative Structural and Computational Biology, The Scripps Research Institute, La Jolla, CA, USA; Department of Integrative Structural and Computational Biology, The Scripps Research Institute, La Jolla, CA, USA; Department of Biochemistry and Molecular Genetics, Center for Health AI, University of Colorado School of Medicine, Aurora, CO, USA; Department of Integrative Structural and Computational Biology, The Scripps Research Institute, La Jolla, CA, USA

## Abstract

Gene definitions and identifiers can be painful to manage–more so when trying to include gene function annotations as this can be highly context-dependent. Creating groups of genes or gene sets can help provide such context, but it compounds the issue as each gene within the gene set can map to multiple identifiers and have annotations derived from multiple sources. We developed MyGeneset.info to provide an API for integrated annotations for gene sets suitable for use in analytical pipelines or web servers. Leveraging our previous work with MyGene.info (a server that provides gene-centric annotations and identifiers), MyGeneset.info addresses the challenge of managing gene sets from multiple resources. With our API, users readily have read-only access to gene sets imported from commonly-used resources such as Wikipathways, CTD, Reactome, SMPDB, MSigDB, GO, and DO. In addition to supporting the access and reuse of approximately 180k gene sets from humans, common model organisms (mice, yeast, etc.), and less-common ones (e.g. black cottonwood tree), MyGeneset.info supports user-created gene sets, providing an important means for making gene sets more FAIR. User-created gene sets can serve as a way to store and manage collections for analysis or easy dissemination through a consistent API.

## Graphical Abstract

**Graphical Abstract:**
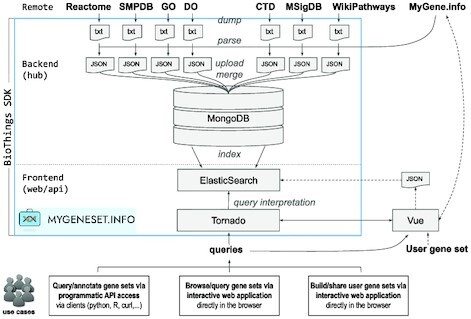
MyGeneset.info provides both programmatic API access and interactive web access to annotated gene sets, either community-curated or user-created.

## INTRODUCTION

Different types of gene-centric information can be accessed through numerous resources, many of which have their own identifiers for accessing information on each gene or gene product. For example, the identifiers for ‘N-glycanase 1’ include 55768 (NCBI Gene) (1), 610661 (OMIM) (2), Q96IV0 (UniProt) (3), etc. Further, each resource may include different annotations describing this gene or gene product such as information on its molecular function (Gene Ontology) (4), chromosomal location (UCSC Genome Browser) (5), clinical significance (ClinGen) (6), and more (7–11). Ensuring up-to-date information on a specific gene can be time-consuming as users would need to either continuously download and merge data from different resources or ensure up-to-date mappings of resource-specific identifiers in their maintenance pipelines.

We have previously reported the creation of MyGene.info, an up-to-date RESTful gene annotation as a service API (Application Programming Interface), which helps simplify the maintenance of pipelines that pull and/or analyze data from gene-specific resources (12,13). However, the functional effects of a gene are often context-dependent, and context can be difficult to capture, let alone make Findable, Accessible, Interoperable, and Reusable (FAIR) (14). A previous server, Tribe, allowed the capturing and sharing of collections of genes (gene sets) (15). However, it supported a subset of identifiers and did not plug into a widely used ecosystem of APIs. To create a FAIR gene-function-focused web server, we use the BioThings SDK (16) to create MyGeneset.info, which supports collaborative, context-storing, user-friendly access to collections of genes both via web and application programming interfaces.

MyGeneset.info can link both software and web-based analytical pipelines. A user might run an analysis on a BioThings-supported webserver (16,17) to generate a set of genes and then access that set through an R analysis. For example, a user could annotate a set of genes that are frequently mutated in a rare cancer. They could then query MyChem.info (https://mychem.info) (16) to identify drugs that target those genes. In parallel, they could analyze RNA-seq data using an R script and then rapidly examine the expression levels of genes within the gene set. Developers of curated resources can use the MyGeneset.info infrastructure to distribute collections within a well-used and highly interoperable ecosystem.

## MATERIALS AND METHODS

### Public gene set data sources

**Figure 1.**
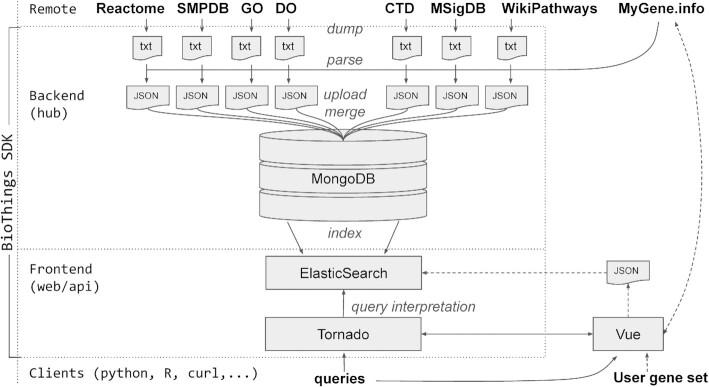
Publicly-accessible, curated gene sets are structured as resource plugins in MyGeneset.info, which was built using the BioThings SDK (dotted box). Each plugin retrieves gene annotations from MyGene.info before uploading and merging the data. Users can submit queries via clients or RESTful queries to the API, or via the Vue-based browser interface. Users can build and submit gene sets via the browser interface (dashed arrows).

MyGeneset.info aggregates multiple publicly available gene sets and provides one-stop access to these built-in, curated gene sets. The current list of data sources, as of March 2023, include CTD (Comparative Toxicogenomics Database) (18), DO (Disease Ontology) (19), GO (Gene Ontology) (4), MSigDB (Molecular Signatures Database) (20), Reactome (7), SMPDB (Small Molecule Pathway Database) (21,22) and WikiPathways (23). There are 188650 (as of February 2023) curated gene sets in total collected from seven data sources. Similar to other biomedical web service APIs we have built (e.g. the MyGene.info API), the BioThings SDK (16) package was used to build a data source ‘hub’ to monitor, download and aggregate underlying data (Figure 1). All curated gene sets and their associated gene information are updated in MyGeneset.info on a weekly to monthly basis based on their respective sources. During each data update, individual gene annotations from each associated gene set are normalized using the MyGene.info API (12,13), with commonly used identifiers included. In the case of retired gene identifiers used in the original data source, it will be either replaced when a gene entity is replaced by a new entity or removed when a gene entity has been retired permanently.

### User submitted gene sets and annotations

MyGeneset.info also allows authenticated users, via their existing Github (https://github.com) or ORCID (24) account, to submit their own gene sets, e.g. from their own research studies or relevant literature. Users may set their gene sets to be either private or public, so that they can be shared when they are ready. The MyGeneset.info web application provides a user interface to help users to annotate and standardize the individual gene annotations within a user gene set using the MyGene.info API. This is the same process by which curated public gene sets are annotated and updated so that users do not need to handle the identifier conversion and maintenance of up-to-date gene annotations.

### Programmatic access via a web-service API

MyGeneset.info provides two primary ways to interact with the service to read and write data: a web-service API and its web application. The API is intended for users who require or prefer programmatic access to the service. The API can be used from a command-line interface or any programming language capable of standard HTTP requests. API access is useful for performing large-scale, batch, or complex analyses in an automated way. Developers may also utilize the API to build web applications that import gene sets and metadata automatically. The same API is powering MyGeneset.info's own web application. The MyGeneset.info API is built upon the BioThings SDK package (16), which allows us to quickly build a set of web API endpoints with rich query features and scalable query performance. The BioThings SDK uses Tornado web framework (http://tornadoweb.org) to create API query endpoints and process the queries passed to an underlying Elasticsearch (https://www.elastic.co/elasticsearch/) cluster, where all gene set data are stored and indexed.

### Web application

The MyGeneset.info web application is intended for users who prefer web-based access to the service. It provides all the same features as the API, but with a user-friendly graphical interface. It allows users to quickly and conveniently search, view and manage gene sets from any connected device. The web application is built as a standard single-page application (SPA) in Vue 3 (https://vuejs.org) and TypeScript (https://www.typescriptlang.org). Its implementation follows modern web development best practices, such as responsive design (i.e. works on desktop and mobile devices) and accessibility, as well as modern Vue-specific best practices, such as using Composition API and script setup syntax.

## RESULTS

### Collecting and sharing publicly available annotated gene sets

**Figure 2.**
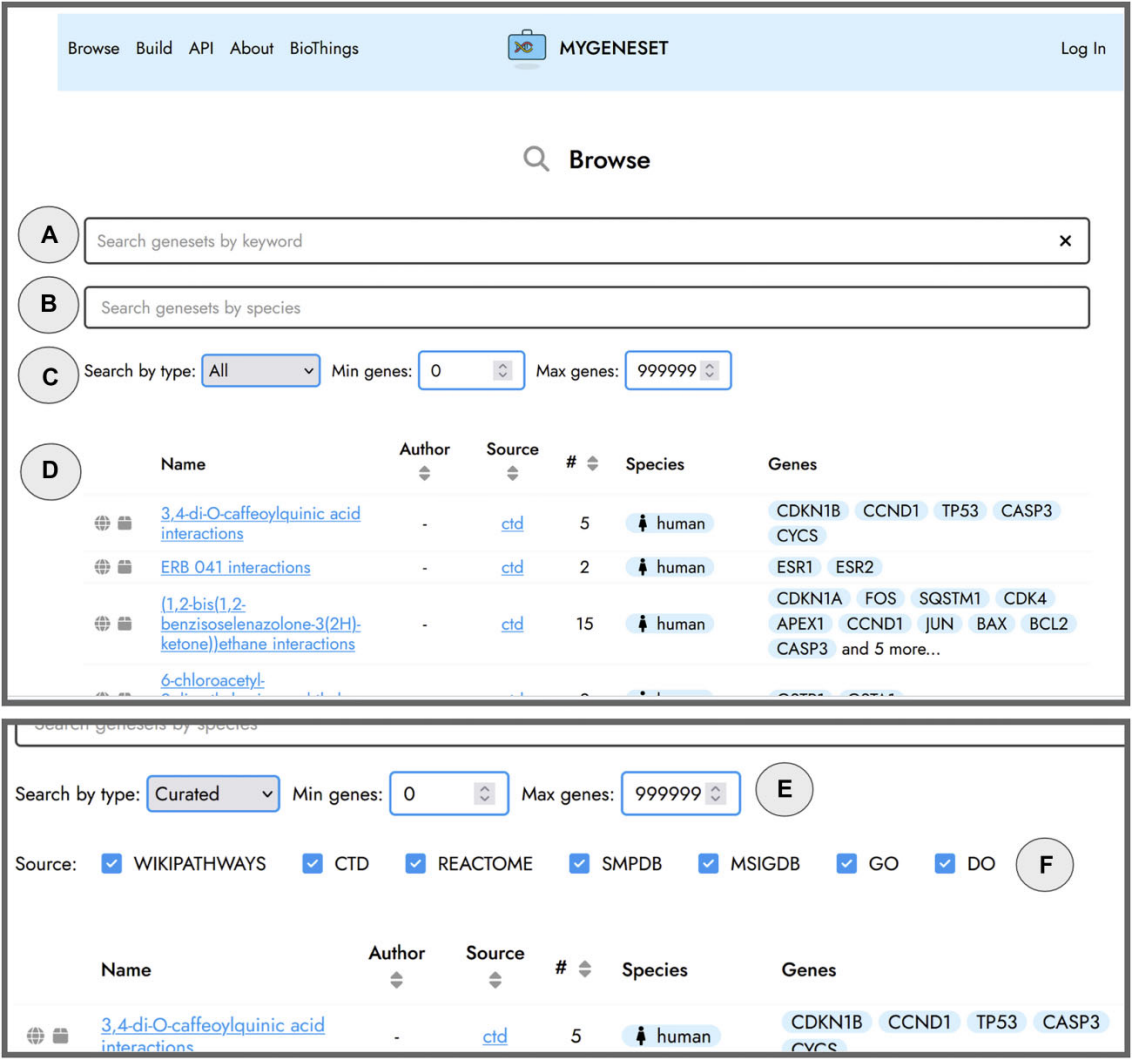
The search and browse interface of MyGeneset.info allows users to search for gene sets by keyword (**A**) or species (**B**). Users can also search by type (**C**) including, gene sets generated by the User, publicly accessible Curated gene sets filterable by source, Anonymously submitted gene sets, and All gene sets. The resulting gene set table can be sorted by author, source, and number of genes in the gene set (**D**). Gene sets can be filtered by the number of genes in the set (**E**) or by the source (**F**) if ‘Curated’ is selected in (C).

MyGeneset.info currently provides read-access for about 180k gene sets, improving the findability and reusability of gene sets from humans, common and uncommon model organisms. As seen in Table 1, different resources have grouped genes into sets based on which chemicals they interact with (CTD), their involvement in specific diseases (DO), their co-localization (GO), their contribution or exclusion in biological processes or molecular functions (GO), their membership in pathways (Reactome, Wikipathways, SMPDB), and more. Users can search for gene sets based on keywords describing the gene or context (Figure 2A), or species from which the gene set was derived (Figure 2B). Although all publicly available gene sets are included by default, the user can also limit their search by type of gene sets to include user-generated gene sets, anonymously submitted gene sets, or select from curated gene sets by source (Figure 2C). The resulting gene sets can be sorted based on the author, source, or number of genes in the gene set (Figure 2D). Users can further filter for gene sets by number of genes in the gene set (Figure 2E) or by source by selecting ‘Curated’ (Figure 2C) and then unchecking sources to be removed from the results (Figure 2F). Hovering over a gene within a gene set will display additional information on the gene including links out to other resources.

**Table 1.** Built-in gene sets from publicly available resources (as of 2023.03.24)

Resource name	URL	license	Gene set type	# of gene sets
Comparative Toxicogenomics Database (CTD)	https://ctdbase.org/	(see site)	chemical interactions	22690
Disease Ontology (DO)	https://disease-ontology.org/	CC0 1.0	disease involvement	4610
Gene Ontology (GO)	http://geneontology.org/	CC BY 4.0	cellular location, biological process, molecular function	183 833
Molecular Signatures Database (MSigDB)	https://www.gsea-msigdb.org/gsea/msigdb	CC BY 4.0	positional, regulatory, cell type and more	33 673
Reactome	https://reactome.org/	CC0 1.0	pathway	2593
WikiPathways	https://www.wikipathways.org/	CC0 1.0	pathway	1711
Small Molecule Pathway Database (SMPDB)	https://smpdb.ca/	(see site)	pathway	48 686

### Collecting and sharing user-annotated gene sets

MyGeneset.info's user-friendly, browser-based gene set builder improves the accessibility of the web server to researchers with less programming experience while ensuring interoperability of the underlying data. The builder page guides users to generate important metadata for making their gene set findable (Supplemental Figure 1A), and researchers must search for genes to add enforcing mapping of the genes included in the user-generated gene set (Supplemental Figure 1B). The interface allows users to search a batch of up to 100 genes at once for inclusion in the gene set. If a user chose to log in before submitting their gene set, the user would be able to edit their gene set later on. Otherwise, a user may submit gene sets without logging in (anonymously), or simply download their gene set locally (if they wish to keep the gene set private) (Supplemental Figure 1C).

### Value proposition of MyGeneset.info for targeted user types

MyGeneset.info was first designed to address the needs of three types of users: biomedical researchers, bioinformatics (core) analysts, and bioinformatics resource developers (Table 2). MyGeneset.info's user-friendly, web browser is meant to improve the findability, accessibility, and re-usability of public gene sets; while the gene set builder is meant to improve the interoperability of user-generated gene sets and provide a GUI-based approach for sharing and maintaining user-generated gene sets. For example, a biomedical researcher may be interested in key genes suspected to be important to a biological process but lack the technical expertise to analyze these genes in the context of high-throughput genomic data. To ensure that their collaborator or bioinformatics core analyzes the correct genes, the researcher can leverage the MyGeneset.info website to search for genes (and filter by an organism of interest), add those genes into a collection, and share that collection either by submitting it to MyGeneset.info or downloading the gene set file in a format acceptable by the collaborator or bioinformatics core. By building the gene set via MyGeneset.info, the gene set will include metadata describing the gene set, an identifier for the gene set itself, and identifiers for each gene in the gene set. Because the gene set builder leverages the MyGene.info API, users can build gene sets from any species with genes annotated by NCBI Gene and Ensembl.

**Table 2.** Feature list and value proposition of each feature for different types of users

	Value of the feature if the user is a:		
Feature	Biomedical researcher	Bioinformatics analyst	Bioinformatics resource developer
User-friendly GUI	Search, create, or share gene sets without the need to program. Easily maintain user-generated gene sets	Remove identifier guess work by having researchers generate machine-friendly gene sets	
Identifier mapping	Ensure each gene in the gene set is mapped to an identifier. Ensure each gene set itself has a referable identifier	Search for gene sets by identifier.	
Built-in gene sets	Find and share existing gene sets	Search for context/gene sets from existing publicly accessible resources	
Gene set creation	Find and add organism-specific genes (with identifiers) into a gene set	Refer less-technical collaborators User-friendly GUI for gene set creation to ensure interoperability	
Gene set download	Download gene sets in machine-friendly format for downstream use by technical collaborators	Find and download gene sets in machine-friendly format for downstream processing	
Gene set submission	Publicly store gene sets for sharing with others. Edit (maintain) submitted gene sets via user-friendly GUI. Make the gene set publicly available with its own identifier	Publicly store pipeline-generated gene sets for sharing with others.	
Gene set API	Refer technical collaborators to API for programmatic access to publicly available gene sets	Programmatically access up-to-date gene sets, off-loading gene set maintenance and storage	Offload gene set and user storage and maintenance

In contrast, a bioinformatics (core) analyst may be tasked with summarizing information for collections of genes from different researchers studying different biological processes in different animal models. Mapping and keeping track of each gene in each collection can be time-consuming, especially if different biomedical researchers provide these gene sets with different types of identifiers (or no identifiers altogether), and in different formats. By having the biomedical researchers use MyGeneset.info, the bioinformatics (core) analyst will be able to receive the gene sets in a consistent, easy-to-read, machine-friendly format either via a file sent from the biomedical researcher or by leveraging the API. For example, if a bioinformatics analyst needs Gene Ontology annotations for all genes in a gene set, they could easily access the gene set via MyGeneset.info's RESTful API using simple python code, pull the identifiers for each gene in the gene set, and then query MyGene.info for additional gene-specific annotations (like GO annotations) (Supplemental Figure 2). Analysts concerned about version changes from the weekly updates of MyGeneset.info can cache the results locally using the BioThings Python client (Supplemental Figure 3).

Similar to a bioinformatics (core) analyst, a bioinformatics resource developer can offload the gene search and identifier mapping of their resource to MyGene.info. With MyGeneset.info, the resource developer can enable users to query for collections of genes, and allow users to create, append, and maintain these collections of genes. Further, if the resource is implemented with OAuth (25), the bioinformatics resource developer could largely offload the implementation of storing and maintaining gene sets and users.

## DISCUSSION AND CONCLUSION

Biomedical researchers are constantly studying the functionality of genes and sets of genes, which require context to interpret; however, sharing context has traditionally been done via free text descriptions (i.e. publications). Given the current volume of scientific literature and its rate of growth, it is difficult to make gene function annotations Findable, Accessible, Interoperable, and Reusable. To do so, some resources have captured contextual information in the form of pathways or as collections of genes. Traditional resources that capture this context (like Wikipathways, CTD, DO and others) improve the FAIRness of gene function annotation by making their annotations available and/or allowing community curation of gene function annotations. Sacrificing some FAIRness for quality, community curation of gene function annotations by different resources have been limited in scope/topic and hampered by the curation process. For example, Wikipathways enables anyone to submit pathway-based gene sets, which may be useful for researchers studying specific pathways, but less useful for researchers studying polygenic diseases where a common pathway has not yet been elucidated. Other resources may curate based on other features but have a lengthier or more restrictive curation process. MyGeneset.info empowers users to share the context of their research on gene function as gene sets, improving the FAIRness of functional gene annotation.

In contrast to community curation, resources have done a phenomenal job making curated gene sets available; however, improvements in FAIRness can still be made. For example Disease Ontology does not directly provide gene sets on its site; rather, DO curates diseases and xrefs to other resources for diseases. The disease-based gene sets generated based on Disease Ontology require traversing from Disease Ontology to OMIM which can be challenging without programmatic expertise. Furthermore, resources that do provide gene sets may use different identifiers for the genes in the gene sets. For example, SMPDB uses UniProt IDs, while Wikipathways uses NCBI Gene IDs. By providing a unified and harmonized resource for gene sets, MyGeneset.info makes it easier to Find gene sets (only have to search one resource as opposed to several), Accessible (availability of a single user-friendly interface for interacting with gene sets), Interoperable (the genes in the gene set are mapped and linked to multiple commonly used identifiers and allow exporting to commonly used identifiers). By improving the FAIRness of gene sets, MyGeneset.info aims to increase their Reusability; thereby making gene sets more FAIR.

MyGeneset.info is integrated with the BioThings API ecosystem (https://biothings.io), which has served >45 million requests in the past 30 days and allows for programmatic traversal from gene sets to genes to variants in those genes or drugs targeting those genes. For example, a user can now easily retrieve a DO-sourced gene set from MyGeneset.info, use the identifiers for the genes in the gene set to pull Gene Ontology annotations from MyGene.info, and perform an overrepresentation analysis using their preferred libraries to investigate processes involved in disease pathology. The user can also use MyGene.info to map the genes to PharmGKB identifiers to find chemicals which might perturb each gene allowing the user to search for compounds that might treat the disease. These compounds could be searched in MyChem.info for additional properties which could affect their appeal as a potential treatment candidate. While not designed for extensive analysis on its own, MyGeneset.info's integration in the BioThings API ecosystem (including the BioThings Python client) makes it suitable for use in downstream analytical workflows or other web servers. Using the client, users can quickly fetch all gene sets available from MyGeneset.info for downstream processing.

MyGeneset.info is fully functional, has documentation, and both the API and front end are fully open source under a permissive Apache 2.0 license. Currently, the gene set creation interface supports the search/upload of up to 100 genes (via the multi-line input mode) in the gene addition process. While this may be sufficient for many biomedical researchers, increasing the batch size and including a means of uploading a list of >100 genes will improve the value of the resource for biomedical researchers and bioinformaticians alike. In the future, we hope to improve the gene set creation process by integrating similarity scores with existing gene sets in the gene set creation process, to minimize redundancy in user-submitted gene sets. Furthermore, we hope to incorporate features for discussing user-submitted gene sets–enhancing collaboration within teams of users.

## DATA AVAILABILITY

The MyGeneset.info web application is available at https://mygeneset.info, and its API can be found at: https://mygeneset.info/api (API documentation at https://docs.mygeneset.info). The source code is available in the GitHub repositories under the Apache 2.0 open-source license: https://github.com/biothings/mygeneset.info (API) and https://github.com/biothings/mygeneset.info-website (web application). Source code files are also on Zenodo: https://doi.org/10.5281/zenodo.7803782 and https://doi.org/10.5281/zenodo.7803811.

## Supplementary Material

gkad289_Supplemental_Files
