# Supplementary material for: MyGeneset.info: an interactive and programmatic platform for community-curated and user-created collections of genes
Source: Nucleic Acids Res. 2023 Apr 18;51(W1):W350–6. doi: 10.1093/nar/gkad289 (PMC10481249; doi:10.1093/nar/gkad289)
Supplement: gkad289_Supplemental_Files [file gkad289_supplemental_files.zip › Supplemental Figures - revision1.pdf]

Supplemental Figure 1- The gene set builder interface

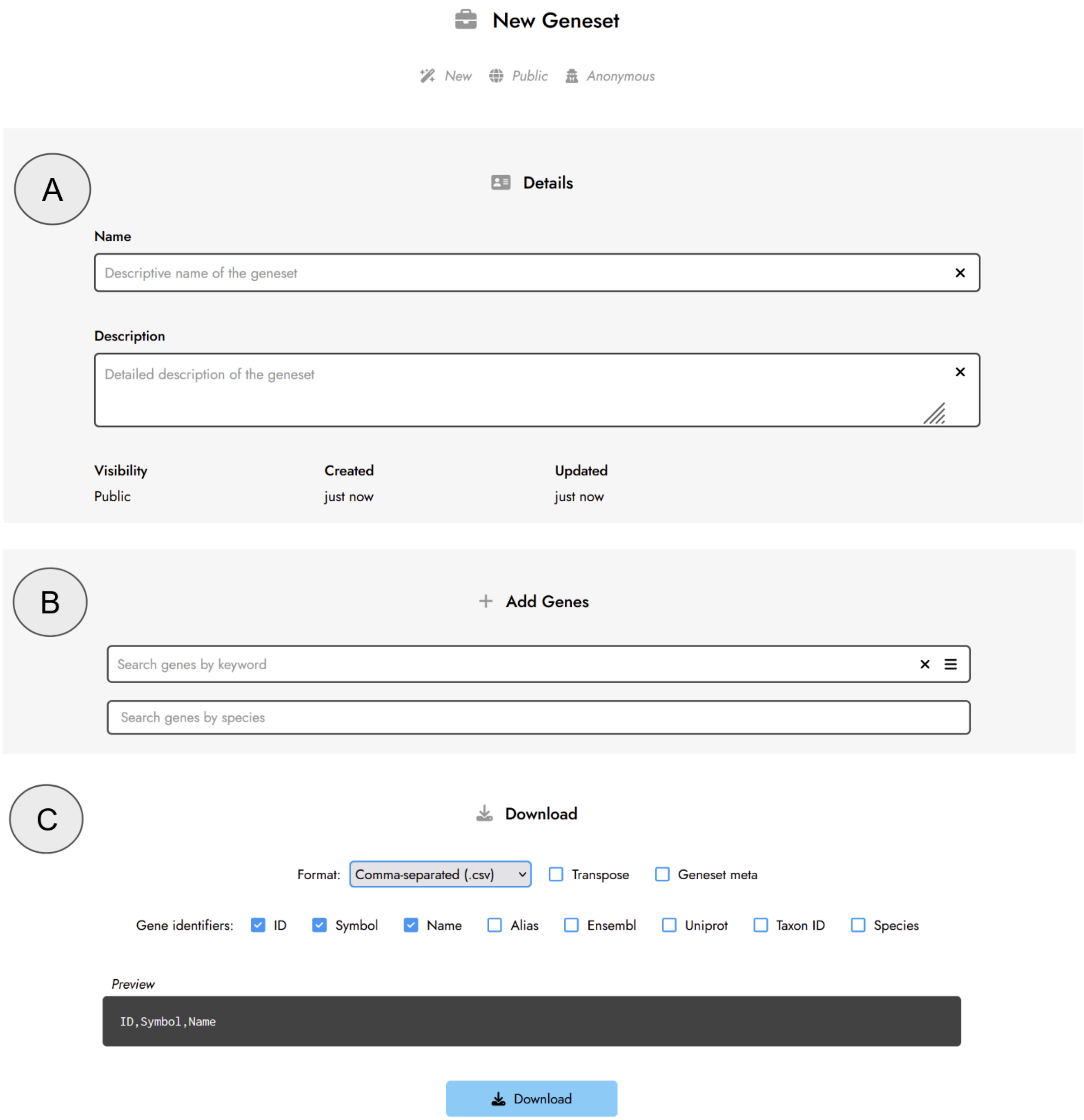

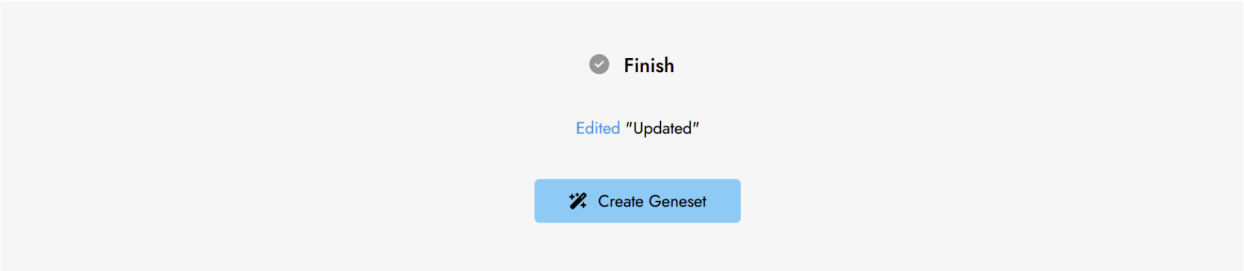

Supplemental Figure 1 - MyGeneset.info's gene set builder encourages users to include descriptive metadata (A) when creating a gene set. Users must search for genes to include, ensuring that each gene included in the gene set is mapped to an identifier (B). Once a user has created a gene set, they can download it in a machine-friendly format to share privately, or submit it (and share it publicly) (C)

Supplemental Figure 2 - Sample code for analytical workflows

This code example with detailed documentation is also available at this Jupyter notebook: https://github.com/biothings/mygeneset.info/blob/master/docs/ipynb/mygeneset_workflow.ipynb

## Fetch gene sets involved in "impaired glucose tolerance", include the taxon id of the gene set itself as well as the name, Uniprot ID, and mygene.info identifier for each gene in the gene set.

```
>>> import requests
>>> from pprint import pprint
>>> r = requests.get(
"https://mygeneset.info/v1/query/?q=%22impaired%20glucose%20toleran
ce%22&fields=taxid,genes.name,genes.uniprot,genes.mygene_id" )
>>> data = r.json()
>>> pprint(data)
```


```
{'took': 3,
 'total': 3,
 'max_score': 37.554295,
 'hits': [
   {'_id': 'HP_IMPAIRED_GLUCOSE_TOLERANCE',
    '_score': 37.554295,
    'Taxid': 9606,
    'genes': [
      {'mygene_id': '10219',
       'name': 'killer cell lectin like receptor G1',
       'uniprot': 'Q96E93'},
      {'mygene_id': '389692',
```

```
        'name': 'MAF bZIP transcription factor A',
```

## For each gene in a gene set, basic identifiers can be retrieved (such as UniProt, NCBI Gene, and others discussed in manuscript) which can be used in compatible downstream applications or to retrieve additional information from compatible resources

```
>>> first_hit = data['hits'][0]
>>> uniprot_ids = [x.get("uniprot", "") for x in
first_hit["genes"]]
>>> # do something with the list of uniprot id
```

## To fetch additional gene information beyond what is stored in MyGeneset.info, we can use the MyGene.info identifiers to leverage the power of MyGene.info. In this manner, we can do a post request to batch fetch the GO annotations for each gene. Other types of available gene-centric annotations and limitations when using MyGene.info can be found in the documentation for the service: https://docs.mygene.info/en/latest/doc/data.html#available-fields)

```
>>> mygene_ids = [x['mygene_id'] for x in first_hit['genes']]
>>> querylist = ','.join(mygene_ids)
>>> params = {"q": querylist, "fields": "name,go"}
>>> res = requests.post("http://mygene.info/v3/query", data=params)
>>> res = res.json()
>>> # do something with the expanded annotations of the gene list
>>> pprint(res[0])
```


```
[
  {
    "query": "10219",
    "_id": "10219",
    "_version": 2,
    "go": {
      "BP": [
        {
          "evidence": "TAS",
          "gocategory": "BP",
          "id": "GO:0006954",
          "pubmed": 9842918,
          "qualifier": "involved_in",
          "term": "inflammatory response"
        },
        {
          "evidence": "TAS",
          "gocategory": "BP",
          "id": "GO:0006968",
```

Supplemental Figure 2 - Python code example illustrating the use of MyGeneset.info for simplifying functional gene set analytical workflows. Users can search for gene sets by keywords and retrieve basic information about the gene set including the name, the NCBI taxon ID of the gene set, common identifiers of gene members etc. The expanded gene-level annotations can be further obtained from the companion MyGene.info API.

Supplemental Figure 3 - Sample code for fetching API metadata with and without the BioThings Python client and caching API calls using the BioThings Python client.

This code example with detailed documentation is also available at this Jupyter notebook: https://github.com/biothings/mygeneset.info/blob/master/docs/ipynb/mygeneset_versioning_caching.ipynb

## Get current build information without the BioThings Python Client

```
>>> import requests
>>> from pprint import pprint
>>> r = requests.get('https://mygeneset.info/v1/metadata/')
>>> data = r.json()
>>> version_date = data['build_date']
>>> pprint(version_date)
```

```
"2023-02-15T10:12:03.921880-08:00"
```

## Installing the universal BioThings client

```
pip install “biothings_client[caching]”
```

Using the universal BioThings client to interact with MyGeneset.info

```
>>> import biothings_client
>>> mgs_client = biothings_client.get_client("geneset")
```

enable the local cache for future API calls

```
>>> local_cache = "mgs_cache"
>>> mgs_client.set_caching(local_cache)
```

Once enabled, any API calls made (like the example call below) will be cached

```
>>> res = mgs_client.querymany(["wnt", "jak-stat"],
fields="name,count,source,taxid")
```

Metadata about the MyGeneset.info API (such as versions, stats, build info) can also be made via the client

```
>>> mgs_metadata = mgs_client.metadata()

# You can stop the caching at the end or anytime you want
mgs_client.stop_caching()
```

Caching can be ended at any time using the client

```
>>> mgs_client.stop_caching()
```

Supplemental Figure 3 - Versioning can be addressed by accessing version information from the metadata endpoint and using the BioThings python client to cache API call responses locally.
