# Supplementary material for: MyGeneset.info: an interactive and programmatic platform for community-curated and user-created collections of genes
Source: Nucleic Acids Res. 2023 Apr 18;51(W1):W350–6. doi: 10.1093/nar/gkad289 (PMC10481249; doi:10.1093/nar/gkad289)
Supplement: gkad289_Supplemental_Files [file gkad289_supplemental_files.zip › mygeneset.info-master.zip!/mygeneset.info-master/docs/_static/widget/autocomplete/demo.html]

Enter a gene here: 


---

## Autocomplete widget for gene query

This Autocomplete widget for gene query provides suggestions while you type a gene symbol or name into the field. Here the gene suggestions are displayed as "<Symbol>:<Name>", automatically triggered when at least two characters are entered into the field.

This widget is powered by "mygene.info" web service. By default, the gene suggestions are limited to human genes only. We will enable more customization options to allow queries against other species soon.

To use this widget in your own website is very easy, just following these three steps:

1. Copy/paste this line into your html file.

```
                <script src="//mygene.info/widget/autocomplete/js/mygene_query_min.js" type="text/javascript"></script>
```

2. Add "mygene\_query\_target" class to your target input element.

```
                <input id="gene_query" style="width:250px" class="mygene_query_target">
```

3. Define your own callback function, which is triggered after user selects a gene.

```
                <script type="text/javascript">
                    mygene_query_select_callback = function(event, ui){
                            alert( ui.item ?
                                "Selected: " + ui.item.label + '('+ui.item._id+')':
                                "Nothing selected, input was " + this.value);
                    };
                </script>
```
