# Supplementary material for: MyGeneset.info: an interactive and programmatic platform for community-curated and user-created collections of genes
Source: Nucleic Acids Res. 2023 Apr 18;51(W1):W350–6. doi: 10.1093/nar/gkad289 (PMC10481249; doi:10.1093/nar/gkad289)
Supplement: gkad289_Supplemental_Files [file gkad289_supplemental_files.zip › mygeneset.info-master.zip!/mygeneset.info-master/docs/_static/widget/autocomplete/demo_plugin.html]

Enter a gene here: 


---

## JQuery UI widget for gene query autocomplete

This autocomplete widget for gene query provides suggestions while you type a gene symbol or name into the field. Here the gene suggestions are displayed as "<Symbol>:<Name>", automatically triggered when at least two characters are entered into the field.

This widget is powered by "mygene.info" web service. By default, the gene suggestions are limited to human genes only. You can customize it to allow queries against other species.

To use this widget in your own website is very easy, just following this example:

```
                <html>
                <head>
                    <link rel="stylesheet" href="//code.jquery.com/ui/1.10.3/themes/ui-lightness/jquery-ui.min.css" type="text/css" media="all" />
                    <script src="//code.jquery.com/jquery-1.9.1.min.js" type="text/javascript"></script>
                    <script src="//code.jquery.com/ui/1.10.3/jquery-ui.min.js" type="text/javascript"></script>
                    <script src="//mygene.info/widget/autocomplete/js/jquery.ui.genequery_autocomplete.js" type="text/javascript"></script>
                </head>
                <body class="ui-widget">
                    <label for="gene_query">Enter a gene here: </label>
                    <input id="gene_query" style="width:250px" class="mygene_query_target">
                    <script type="text/javascript">
                        $('input.mygene_query_target').genequery_autocomplete();
                    </script>
                </body>
                </html>
```

To define your own callback function:

```
                    <script type="text/javascript">
                        $('input.mygene_query_target').genequery_autocomplete({
                            select: function(event, ui) {
                                        alert( ui.item ?
                                            "Selected: " + ui.item.label + '('+ui.item._id+')':
                                            "Nothing selected, input was " + this.value);
                                    }
                        });
                    </script>
```

To customized this widget to fit your need, you can refer to options/events listed for the original JQueryUI autocomplete widget. Besides them, these are some options specific to this widget only (with their default values):

```
                {
                    "mygene_url": "//mygene.info/v3/query",
                    "q": "{term}",
                    "species": "human",
                    "fields": "name,symbol,taxid,entrezgene",
                    "limit": 20,
                    "gene_label": "{symbol}: {name}",
                    "value_attr": 'symbol',
                }
```
