# Supplementary material for: MyGeneset.info: an interactive and programmatic platform for community-curated and user-created collections of genes
Source: Nucleic Acids Res. 2023 Apr 18;51(W1):W350–6. doi: 10.1093/nar/gkad289 (PMC10481249; doi:10.1093/nar/gkad289)
Supplement: gkad289_Supplemental_Files [file gkad289_supplemental_files.zip › mygeneset.info-master.zip!/mygeneset.info-master/docs/_templates/layout.html]

{# Import the theme's layout. #}
{% extends "!layout.html" %}
{# Include our new CSS file into existing ones. #}
{% set css\_files = css\_files + ['\_static/mgdocs-styles.css']%}
{%- block extrahead %}
{{ super() }}

{% endblock %}
